# General self-efficacy in East and West Germany: A comparison of two German representative cohorts in 2014 and 2022

**DOI:** 10.1016/j.puhip.2025.100718

**Published:** 2025-12-18

**Authors:** Anna C. Reinwarth, Julia Petersen, Manfred E. Beutel, Kerstin Weidner, Vera Clemens, Elmar Brähler

**Affiliations:** a Department of Psychiatrie and Psychotherapy, University Medical Center of the Johannes Gutenberg-University Mainz, Mainz, Germany; b Department of Psychosomatic Medicine and Psychotherapy, University Medical Center of the Johannes Gutenberg-University Mainz, Mainz, Germany; c Department of Psychotherapy and Psychosomatic Medicine, Faculty of Medicine, Technische Universität Dresden, Dresden, Germany; d Department for Child and Adolescent Psychiatry/Psychotherapy, University of Ulm, Ulm, Germany; e Department of Psychiatry and Psychotherapy, Medical Faculty, University of Leipzig, Leipzig, Germany

**Keywords:** General self-efficacy, East Germany, West Germany, Sex-differences

## Abstract

**Objectives:**

Empirical evidence increasingly highlights the importance of general self-efficacy (GEF) in preventing disease and promoting quality of life. While it is already known that GEF varies with socio-demographic factors, health variables and personality traits, little is known about the influence of socio-political context. The objective of the study was to examine and compare GEF between 2014 and 2022 in East and West Germany and to test differences regarding sex.

**Study design:**

In 2014 (N = 2506) and 2022 (N = 2508), two large representative cohorts from the German general population were surveyed about their GEF using the General Self-Efficacy Short Scale (ASKU).

**Methods:**

A three-way ANCOVA were calculated to test the effect of region of residence, survey date, and sex controlling for age on GEF in a combined sample (N = 5014).

**Results:**

There was a significant decrease in GEF between 2014 and 2022. Women reported lower GEF than men. A statistically significant interaction was found between survey date and sex and between survey date, region of residence and sex on GEF. Almost the same patterns were observed for the ability to solve difficult and complex tasks well, with the exception, that men in East Germany reported an increase in the ability to solve difficult and complex tasks well from 2014 to 2022. The ability to solve most problems independently and to solve challenging and complex tasks well was mainly influenced by education and household income, rather than by the date of the survey, the region of residence or sex.

**Conclusion:**

Although regional differences in GEF were minimal, more pronounced variations emerged across sex and socioeconomic groups. These patterns likely stem from historical socio-political legacies and structural inequalities, potentially amplified by the impact of COVID-19 restrictions.

**What this study adds:**

- Insights into how socio-political context influences general self-efficacy (GEF)
- Results regarding the development of GEF from 2014 to 2022
- Identification of sex-based patterns

**Implications for Policy and Practice:**

- No significant overall differences emerged between East and West Germany
- Observed distinct sex-based variations within each region should be taken seriously
- Women tended to report significantly lower GEF, highlighting the need for gender-equal political approaches in the future

## 1 Introduction

Self-efficacy refers to an individual's belief of achievement, productivity and competence in daily life [1]. Self-efficacy has profound effects on human behaviour and well-being [2]. While a high level of self-efficacy leads to a proactive mindset, fosters perseverance in difficult situations, and increases motivation to persist in achieving goals, even in the face of obstacles, a lack of self-efficacy can result in feelings of helplessness and powerlessness, reducing the willingness to face new challenges [3]. The generalised belief in one's own capabilities to produce given attainments in various situations and areas of activity is defined as general self-efficacy (GEF) [4].

To date, a growing body of empirical evidence increasingly highlights the importance of GEF in preventing disease [5] and promoting mental health [[6], [7], [8]]. Further, it is positively associated with quality of life [9]. Moreover, the recent COVID-19 pandemic showed that those with higher levels of self-efficacy were generally more resilient to the stressful negative effects on mental health [10].

GEF varies among socio-demographic factors and personality traits. A recent longitudinal study of the UK suggested that GEF was positively associated with age, education and occupation, cognitive ability, and personality traits and negatively with female sex [11]. Furthermore, a meta-analysis of 187 studies observed gender differences in academic self-efficacy with women generally reporting lower levels in self-efficacy, especially in the domains of mathematics, computer, and social sciences [12].

Germany offers a compelling case for examining macro-level influences on general self-efficacy (GSE), shaped by its distinct socio-political history. From 1949 to 1990, the country was split into two ideologically opposed states: the capitalist, democratic Federal Republic of Germany (FRG) in the West and the socialist German Democratic Republic (GDR) in the East. These divergent systems created fundamentally different societal structures, economic models, and socialization patterns. For instance, the GDR promoted full female labour participation through extensive childcare and state policies, while the FRG maintained a more traditional “male breadwinner” model with limited support for working mothers.

Reunification in 1990 brought major economic disruption, especially in the East. Rapid privatization led to widespread company closures and mass unemployment, as former GDR firms struggled to compete [13]. These structural shocks left lasting effects: eastern states still lag in income, wealth, and employment. East Germans report higher perceived deprivation and lower life satisfaction than West Germans [14,15], alongside regional differences in mental health and psychological well-being, particularly in the years following reunification [[14], [15], [16]].

Migration patterns have further shaped disparities. After reunification, many young, educated East Germans moved westward, deepening demographic and economic imbalances [17]. Since around 2017, however, some eastern regions have seen net in-migration from the West, driven by lower living costs, better infrastructure, and growing economic opportunities [18]. These dynamics likely influence both individual and collective levels of self-efficacy across regions.

### 1.1 Aim

Given the importance of GEF for health maintenance and prevention, this study examines and compares GEF between 2014 and 2022 in East and West Germany using data from two large representative cohorts of the German general population. Given the socio-economic differences between East and West Germans, comparing the two regions in terms of GEF may allow conclusions to be drawn about the consequences of two socio-political systems in the past. Furthermore, sex differences are tested in a pseudo longitudinal design.

## 2 Methods

### 2.1 Study Design and participants

For the present study, data from two representative German population surveys were used. The first survey (survey A) was conducted in February to April 2014 (N = 2527) and the second one (survey B) in March to May 2022 (N = 2522) by the independent market research institute USUMA using the same methodology. For a description of the procedure, see supplementary material S1a/b.

For our analyses, we excluded participants with missing data for GEF in both surveys (n = 21 in survey A, n = 10 in survey B). Due to a lack of representativeness and the absence of a comparable figure in Survey A, missing values were also assigned to a small number of Survey B participants who did not identify as male or female (n = 4), leading to a final analysis sample of N = 2506 of survey A and a final analysis sample of N = 2508 of survey B.

### 2.2 Measures

#### 2.2.1 Sociodemographic characteristics

Sociodemographic characteristics were assessed in a structured interview. For our analysis, we included data on age, sex (male/female), the highest achieved school degree (recoded in 0 = no high school degree and 1 = having a high school degree), household income per month (1= < 1250 euro, 2 = 1250 - < 2500 euro, 3 = > 2499 euro), and region of residence (East Germany/West Germany).

#### 2.2.2 General self-efficacy

To determine GEF, the General Self-Efficacy Short Scale (ASKU) [1] was used. The ASKU is a short version of the 10-item General Self-Efficacy Scale developed by Schwarzer and Jerusalem [19] (convergent validity r = 0.75) and captures beliefs about one's ability to achieve goals and complete tasks with three items on a 5-point Likert scale (1 = “does not apply at all” to 5 = “applies completely”) [1]. With a McDonald's omega ranging between 0.81 and of 0.86 in a recent representative study of the German population, the internal consistency can be considered excellent [20]. The average of the items for the ASKU was used as the aggregated score in the analysis. The average scale score varies between 1 and 5 with higher scores indicating higher levels of self-efficacy.

### 2.3 Statistical analyses

Descriptive statistics including absolute numbers with percentages and means with standard deviations of socio-demographic characteristics were calculated for each cohort stratified by region of residence (East and West Germany). Further, *t*-test for continuous and chi-square tests for categorical variables were used to analyse differences of distribution and means between the two regions of residence. A three-way ANCOVA (including post-hoc analyses) was calculated to test the effect of region of residence (East/West), survey date (2014/2022) and sex (male/female) on GEF controlling for age, education and household income using a pseudo longitudinal sample combining the two cross-sectional samples of 2014 and 2022 (N = 5014). Main effects and interactions between survey date, region of residence and sex were analysed. Before carrying out the ANCOVA, the homogeneity of variance, the normality of the residuals and the homogeneity of the regression slopes were tested. Results of the tests are shown in supplementary material S4a-d to S6.

For the ANCOVA, age was centred on the mean. For all test procedures, a significance level of *p* < .05 was chosen. All calculations were computed using SPSS version 29.

## 3 Results

### 3.1 Sample description

In the 2014 cohort, the mean age of the participants was 49.43 years (SD = 17.85). Of the 2506 participants, 1341 (53.51 %) were women and 501 (19.99 %) lived in East Germany. Participants interviewed in 2022 (N = 2508) had a mean age of 49.29 years (SD = 17.68), 1260 (50.24 %) were women. 532 (21.21 %) participants lived in East Germany. The mean age of the combined sample (N = 5014) was 49.36 years (SD = 17.76), of whom 2601 (51.87 %) were women and 1033 (20.60 %) lived in East Germany. See Table 1 for a detailed socio-demographic description of the two cohorts.

**Table 1:** Socio-demographic characteristics of the two cohorts (total and stratified by region of residence).

	2014							2022						
	Total (N = 2506)		West (n = 2005)		East (n = 501)		p-value	Total (N = 2508)		West (n = 1976)		East (n = 532)		p-value
Age (M, Sd)	49.43	17.85	48.72	17.67	52.26	18.27	<0.001	49.29	17.68	49.06	17.47	50.16	18.40	0.218
Sex (n, %)							0.968							<. 001
Male	1165	46.48	933	46.53	232	46.30		1248	49.76	922	46.66	326	61.28	
Female	1341	53.51	1072	53.47	269	53.69		1260	50.24	1054	53.34	206	38.72	
High school degree (n, %)							0.163							0.670
Yes	488	19.47	402	20.05	86	17.17		619	24.72	492	24.94	127	23.92	
No	2018	80.53	1603	79.95	415	82.83		1885	75.28	1481	75.06	404	76.08	
Household income (n, %)							<0.001							<0.001
< 1250 euros	457	18.76	314	16.13	143	29.24		225	9.09	184	9.47	41	7.71	
1250 to 2500 euros	1067	43.80	840	43.14	227	46.42		962	38.88	718	36.97	244	45.86	
> 2500 euros	912	37.44	793	40.73	119	24.34		1287	52.02	1040	53.55	247	46.43	

***Note.****M* = Mean; *SD* = Standard deviation; p - values derived from chi-square and t-tests.

### 3.2 GEF in East and West Germany in 2014 and 2022

A three-way ANCOVA examined the effects of survey date, region of residence, and sex on GEF as measured by the ASKU and its three items. Significant effects emerged for survey date (F(1, 4894) = 5.13, p = .023, *η*_*p*_^*2*^ < 0.01) and sex (F(1, 4894) = 5.98, p = .012, *η*_*p*_^*2*^ = 0.01), but not for region. Significant interactions were found between survey date and sex (F(1, 4894) = 4.63, p = .031, *η*_*p*_^*2*^< 0.01) and between survey date, region, and sex (F(1, 4894) = 4.59, p = .010, *η*_*p*_^*2*^ < 0.01). Mean GEF was lower in 2022 (M = 3.95 ± 0.77) than in 2014 (M = 4.06 ± 0.81). Women reported lower GEF overall (M = 3.92 ± 0.79) than men (M = 4.10 ± 0.79). Men showed a greater decline (2014: M = 4.16 ± 0.81; 2022: M = 4.04 ± 0.77) than women (2014: M = 3.97 ± 0.80; 2022: M = 3.87 ± 0.77). Among men, the decline was stronger in West Germany (2014: M = 4.15 ± 0.80; 2022: M = 4.01 ± 0.77) than in East Germany (2014: M = 4.17 ± 0.86; 2022: M = 4.12 ± 0.76). For women, the opposite pattern appeared; those in the West showed a smaller decline (2014: M = 3.95 ± 0.81; 2022: M = 3.88 ± 0.77) than those in the East (2014: M = 4.03 ± 0.78; 2022: M = 3.80 ± 0.78). Significant main effects were also found for education (F(1, 4894) = 38.46, p < .001, *η*_*p*_^*2*^ = 0.01) and household income (F(1, 4894) = 184.96, p < .001, *η*_*p*_^*2*^ = 0.04), as well as an interaction between survey date and age (F(1, 4894) = 6.69, p = .010, *η*_*p*_^*2*^ < 0.01). Participants without a high school degree reported lower GEF (M = 3.95 ± 0.81) than those with a degree (M = 4.20 ± 0.72), and lower income was also associated with lower GEF (1: M = 3.65 ± 0.92; 2: M = 3.95 ± 0.80; 3: M = 4.16 ± 0.71).

#### 3.2.1 Item 1: “I can rely on my own abilities in difficult situations”

No significant main effects of survey date, region of residence, or sex on Item 1 were found (all *p* > .05). Likewise, there were no significant interaction effects between these three factors (all *p* > .05). In contrast, there were significant main effects of the covariates education (F (1, 4894) = 27.76, *p* < .001, *η*_*p*_^*2*^ = 0.01, and household income (F (1, 4894) = 163.97, *p* < .001, *η*_*p*_^*2*^ = 0.03). Further, a significant interaction between survey date and age on Item 1 was found (F (1, 4894) = 5.04, *p* = .025, *η*_*p*_^*2*^ < 0.01). Participants with no high school degree of the combined sample were less likely to report that they can rely on their own abilities in difficult situations (M = 4.00 ± 0.85) than participants with high school degree (M = 4.22 ± 0.80). A lower income was also associated with less agreement with Item 1 ((1: M = 3.70 ± 0.95; 2: M = 4.00; SD = 0.84; 3: M = 4.19 ± 0.78)).

#### 3.2.2 Item 2: “I am able to solve most problems on my own”

No significant main effects of survey date, region of residence, or sex on Item 2 were found (all *p* > .05). Likewise, there were no significant interaction effects between these three factors (all *p* > .05). In contrast, there were significant main effects of the covariates education (F (1, 4895) = 18.93, *p* < .001, *η*_*p*_^*2*^ = 0.01, and household income (F (1, 4895) = 134.58, *p* < .001, *η*_*p*_^*2*^ = 0.03). Participants with no high school degree of the combined sample were less likely to report solving most problems on their own (M = 3.99 ± 0.86) than participants with high school degree (M = 4.19 ± 0.79). A lower income was also associated with less agreement with Item 2 ((1: M = 3.71 ± 0.96; 2: M = 3.99; SD = 0.86; 3: M = 4.17 ± 0.77)).

#### 3.2.3 Item 3: “I can usually solve even challenging and complex tasks well”

Responses to Item 3 showed significant effects of survey date (F(1, 4894) = 7.09, p = .008, *η*_*p*_^*2*^ < 0.01) and sex (F(1, 4894) = 7.91, p = .005, *η*_*p*_^*2*^ = 0.01). Significant interactions were found between region and sex (F(1, 4894) = 4.23, p = .040, *η*_*p*_^*2*^ < 0.01), survey date and sex (F(1, 4894) = 7.30, p = .007, *η*_*p*_^*2*^ < 0.01), and survey date, region, and sex (F(1, 4894) = 6.35, p = .012, *η*_*p*_^*2*^ < 0.01).

Participants in 2022 were less likely to report being able to solve challenging and complex tasks well (M = 3.91 ± 0.87) than in 2014 (M = 3.97 ± 0.92). Men reported higher scores overall (M = 4.03 ± 0.88) than women (M = 3.85 ± 0.90) and showed a greater decline between 2014 (M = 4.07 ± 0.90) and 2022 (M = 3.99 ± 0.87) than women (2014: M = 3.87 ± 0.92; 2022: M = 3.83 ± 0.87). Among women, the decline was stronger in East Germany (2014: M = 3.89 ± 0.93; 2022: M = 3.70 ± 0.91) than in the West (2014: M = 3.87 ± 0.91; 2022: M = 3.86 ± 0.86). East German men slightly increased their ratings (2014: M = 4.03 ± 0.96; 2022: M = 4.06 ± 0.87), whereas West German men showed a decrease (2014: M = 4.09 ± 0.88; 2022: M = 3.96 ± 0.86).

Significant main effects were also observed for education (F(1, 4894) = 53.16, p < .001, *η*_*p*_^*2*^ = 0.02) and household income (F(1, 4894) = 166.35, p < .001, *η*_*p*_^*2*^ = 0.03), along with interactions between survey date and age (F(1, 4894) = 9.49, p = .001, *η*_*p*_^*2*^ < 0.01) and between sex and age (F(1, 4894) = 5.86, p = .016, *η*_*p*_^*2*^ < 0.01). Participants without a high school degree reported lower self-efficacy on this item (M = 3.87 ± 0.91) than those with a degree (M = 4.19 ± 0.78), and lower income was likewise associated with lower scores (1: M = 3.56 ± 1.04; 2: M = 3.87 ± 0.89; 3: M = 4.12 ± 0.81).

Table 2 shows the results with regard to ASKU and item 1 to 3. Supplementary material S7a-l presents the results of the post-hoc analyses investigating the impact of our main variables (region of residence, survey date and sex).

**Table 2:** Results of the three-way ANCOVA on general self-efficacy (measured by the ASKU) and its three items.

	Sum of squares	*df*	F - value	95 % CI	p - value	*η*_*p*_^*2*^
**ASKU**						
**Effect**						

region of residence	0.33	1	0.56	−0.75; 0.33	0.454	<0.01
survey date	3.00	1	5.13	−0.92; −0.07	**.023**	<0.01
sex	3.45	1	5.98	−0.96; −0.10	**.012**	0.01
age	1.28	1	2.18	−0.01; 0.00	0.140	0.01
education	22.48	1	38.46	0.11; 0.22	**< .001**	0.01
household income	108.12	1	184.96	0.19; 0.26	**< .001**	0.04
region of residence * survey date	1.19	1	2.03	−0.09; 0.58	0.154	<0.01
region of residence * sex	1.71	1	2.93	−0.04; 0.64	0.087	<0.01
survey date * sex	2.71	1	4.63	0.03; 0.57	**.031**	<0.01
survey date * age	3.91	1	6.69	0.00; 0.01	**.010**	<0.01
sex * age	2.17	1	3.71	−0.00; 0.00	0.054	<0.01
region of residence * survey date * sex	2.61	1	4.46	−0.44; −0.02	**.035**	<0.01

**Item 1**						
**Effect**						

region of residence	0.00	1	0.03	−0.63; 0.53	0.860	<0.01
survey date	2.50	1	3.75	−0.91; 0.01	0.053	0.01
sex	1.90	1	2.86	−0.86; 0.06	0.091	0.01
age	1.10	1	1.62	−0.01; 0.00	0.202	0.01
education	18.60	1	27.76	0.10; 0.21	**< .001**	0.01
household income	110.10	1	163.97	0.19; 0.26	**< .001**	0.03
region of residence * survey date	0.60	1	0.93	−0.18; 0.53	0.336	<0.01
region of residence * sex	0.70	1	1.11	−0.17; 0.56	0.292	<0.01
survey date * sex	1.40	1	2.15	−0.07; 0.51	0.142	<0.01
survey date * age	3.40	1	5.04	0.00; 0.01	**.025**	<0.01
sex * age	1.70	1	2.61	−0.00; 0.00	0.107	<0.01
region of residence * survey date * sex	1.40	1	2.04	−0.40; 0.06	0.153	<0.01

**Item 2**						
**Effect**						

region of residence	0.20	1	0.29	−0.74; 0.43	0.593	<0.01
survey date	1.80	1	2.63	−0.85; 0.08	0.105	<0.01
sex	3.10	1	4.60	−0.97; −0.04	0.032	0.01
age	0.00	1	0.04	−0.00; 0.00	0.846	0.01
education	12.90	1	18.93	0.07; 0.18	**< .001**	0.01
household income	91.70	1	134.58	0.17; 0.24	**< .001**	0.03
region of residence * survey date	0.70	1	1.03	−0.17; 0.55	0.310	<0.01
region of residence * sex	1.70	1	2.47	−0.07; 0.66	0.116	<0.01
survey date * sex	2.00	1	2.92	−0.04; 0.55	0.087	<0.01
sex * age	1.10	1	1.65	−0.00; 0.00	0.200	<0.01
region of residence * survey date * sex	2.20	1	2.30	−0.44; 0.02	0.069	<0.01

**Item 3**						
**Effect**						

region of residence	1.40	1	1.83	−1.03; 0.19	0.176	<0.01
survey date	5.30	1	7.09	−1.14; −0.17	**.008**	<0.01
sex	5.90	1	7.91	−1.18; −0.21	**.005**	0.01
age	2.10	1	2.86	−0.01; 0.00	0.091	0.02
education	39.50	1	53.16	0.16; 0.28	**< .001**	0.02
household income	123.50	1	166.35	0.20; 0.28	**< .001**	0.03
region of residence * survey date	2.80	1	3.74	−0.01; 0.75	0.053	<0.01
region of residence * sex	3.10	1	4.23	0.02; 0.78	**.040**	<0.01
survey date * sex	5.40	1	7.30	0.12; 0.73	**.007**	<0.01
survey date * age	7.00	1	9.49	0.00; 0.01	**.002**	<0.01
sex * age	4.40	1	5.86	−0.01; −0.00	**.016**	<0.01
region of residence * survey date * sex	4.70	1	6.35	−0.55; −0.07	**.012**	<0.01

***Note.****df* = degrees of freedom; 95 % CI = 95 % confidence interval; *η*_*p*_^*2*^ = partial eta-squared; significant values are printed in bold.

## 4 Discussion

The important role of GEF for physical and mental health is increasingly recognized e.g., Refs. [5,6]. Thus, the present study analysed and compared GEF between 2014 and 2022 in former East and West Germany in a pseudo longitudinal design using data from two large representative cohorts of the German general population. Moreover, differences between women and men were tested.

A three-way ANCOVA examined the effects of survey date, region of residence, and sex on general self-efficacy. Significant effects were found for survey date and sex: participants reported lower GEF in 2022 than in 2014. This decline may reflect the impact of the COVID-19 pandemic and its restrictions, which limited personal control. Supporting this, studies found reduced self-efficacy during the pandemic [21]. Economic consequences of inflation and the Ukraine war in 2022 may have further weakened general economic self-efficacy, as financial strain and poverty are linked to lower self-efficacy [22]. In fact, low household income was constantly linked to lower levels of self-efficacy. A decline in income may, therefore, likely impact self-efficacy.

Furthermore, women consistently reported significantly lower GEF than men in both 2014 and 2022, aligning with previous research [12]. Women, especially in male-dominated fields and academia, often feel less self-effective due to gendered socialization. Interestingly, among East Germans in 2014, sex differences appeared only for Item 1 (“I can rely on my own abilities in difficult situations”), suggesting minimal gender gaps in self-efficacy – likely reflecting the former GDR's more egalitarian gender norms. By 2022, however, significant sex differences emerged for all items and total scores, possibly indicating an adaptation to West German values, where women have consistently reported lower self-efficacy. Prior studies also note that once-egalitarian gender beliefs in the East have shifted toward more traditional views, particularly among younger generations [23].

We also found a significant interaction between survey date, region, and sex. While West German men showed a greater decline in GEF between 2014 and 2022 than East German men, West German women's decline was smaller than that of East German women. The decline among West German men may stem from pandemic-related restrictions, which challenged a group generally less exposed to everyday constraints. In contrast, the sharper decline among East German women may reflect the aforementioned re-traditionalization of gender roles.

Contrary to expectations, no significant overall GEF differences appeared between East and West Germany. However, East Germans scored higher on Item 2 (“I can rely on my own abilities in difficult situations”), possibly reflecting a historically rooted solidarity and self-reliance developed during years of material scarcity and social cooperation. This finding may also indicate that patterns of GEF differences are more nuanced, reflecting sex- and socioeconomic variations within regions rather than clear-cut East–West divides.

### 4.1 Strengths and limitations

Though this study benefitted from large, representative samples of the German population, several limitations must be acknowledged. First, the cross-sectional design limits causal interpretation – we cannot determine whether the observed associations reflect true causal relationships. Second, as self-reported data were used, social desirability bias may have influenced responses. Third, the second survey was conducted during the COVID-19 pandemic, which may have affected self-efficacy levels; given the design, we cannot disentangle pandemic-related effects from genuine temporal changes. In addition, only a limited set of covariates was available, restricting control for confounding factors, and participants’ geographical origins were not assessed, meaning some respondents in the West may have been socialized in the East and vice versa. Finally, the effect sizes of the mean difference tests were very small, suggesting that while some effects reached statistical significance, their practical relevance should be interpreted with caution. Future research should address these limitations.

### 4.2 Conclusion

This study examined self-efficacy differences between East and West Germans in 2014 and 2022, while also considering potential sex differences. While no significant overall differences emerged between East and West Germany, we observed nuanced sex- and socioeconomically based variations within each region, hinting at a more individual-level impact on self-efficacy. This finding may help in clinical contexts, as cognitive therapy may be able to positively affect self-efficacy. Further longitudinal research may investigate these associations in more depth.

## Availability of data and materials

The datasets supporting the conclusions of this article are available from the corresponding author on reasonable request.

## Ethical statement

Prior to the study enrolment, detailed information about the procedures, data collection, and anonymization of personal data were given to the potential participants. Then, verbal informed consent to participate in the study was obtained and confirmed by the interviewers. The studies contents and procedures were approved by the Ethics Commission of the Medical Faculty of the University Leipzig (survey A: 063‐14‐10032014, survey B: 594/21‐ek). Both surveys were conducted in accordance to the ICH-GCP-guidelines and the ICC/ESOMAR International Code of Marketing and Social Research Practice. They were also conducted in accordance with the principles expressed in the Declaration of Helsinki.

## Funding

This research did not receive any specific grant from funding agencies in the public, commercial, or not-for-profit sectors.

## Declaration of competing interest

The authors declare that they have no competing interests.

## References

[1] Beierlein C., Kovaleva A., Kemper C.J., Rammstedt B. (2012).

[2] Bandura A. (1977). Self-efficacy: toward a unifying theory of behavioral change. Psychol. Rev..

[3] Bandura A. (1997).

[4] Bandura A. (2006). Guide for constructing self-efficacy scales. Self-Efficacy Beliefs Adolescents.

[5] Gangwani R., Cain A., Collins A., Cassidy J.M. (2022). Leveraging factors of self-efficacy and motivation to optimize stroke recovery. Front. Neurol..

[6] Djourova N.P., Rodríguez Molina I., Tordera Santamatilde N., Abate G. (2020). Self-efficacy and resilience: mediating mechanisms in the relationship between the transformational leadership dimensions and well-being. J. Leader. Organ Stud..

[7] Meyer N., Niemand T., Davila A., Kraus S. (2022). Biting the bullet: when self-efficacy mediates the stressful effects of COVID-19 beliefs. PLoS One.

[8] Volz M., Voelkle M.C., Werheid K. (2019). General self-efficacy as a driving factor of post-stroke depression: a longitudinal study. Neuropsychol. Rehabil..

[9] Selzler A.-M., Habash R., Robson L., Lenton E., Goldstein R., Brooks D. (2020). Self-efficacy and health-related quality of life in chronic obstructive pulmonary disease: a meta-analysis. Patient Educ. Counsel..

[10] Steigleder J., Buhr L., Ehm J.-H., Gawrilow C., von Suchodoletz A. (2023). Changes in subjective stress experiences and self-efficacy beliefs of preschool teachers in Germany: a longitudinal study during 12 months of the COVID-19 pandemic. Teach. Teach. Educ..

[11] Furnham A., Cheng H. (2024). The big-five personality factors, cognitive ability, health, and social‐demographic indicators as independent predictors of self‐efficacy: a longitudinal study. Scand. J. Psychol..

[12] Huang C. (2013). Gender differences in academic self-efficacy: a meta-analysis. Eur. J. Psychol. Educ..

[13] Martens B. (2020).

[14] Beutel M.E., Krakau L., Schmutzer G., Brähler E. (2021). Somatic symptoms in the Eastern and Western states of Germany 30 years after unification: population-based survey analyses. J. Psychosom. Res..

[15] Bramesfeld A., Grobe T., Schwartz F.W. (2010). Prevalence of depression diagnosis and prescription of antidepressants in East and West Germany: an analysis of health insurance data. Soc. Psychiatr. Psychiatr. Epidemiol..

[16] Ihle W., Esser G., Schmidt M.H., Blanz B., Reis O., Meyer-Probst B. (2001). Prevalence, course, and risk factors for mental disorders in young adults and their parents in East and West Germany. Am. Behav. Sci..

[17] Fuchs‐Schündeln N., Schündeln M. (2009). Who stays, who goes, who returns? East–West migration within Germany since reunification 1. Econ. Transit..

[18] Stawarz N., Sander N., Sulak H., Rosenbaum-Feldbrügg M. (2020). The turnaround in internal migration between East and West Germany over the period 1991 to 2018. Demogr. Res..

[19] Schwarzer R., Jerusalem M. (1999).

[20] Weidner K., Bittner A., Beutel M., Goeckenjan M., Brähler E., Garthus-Niegel S. (2020). The role of stress and self-efficacy in somatic and psychological symptoms during the climacteric period–Is there a specific association?. Maturitas.

[21] Pressley T., Ha C. (2021). Teaching during a pandemic: united States teachers' self-efficacy during COVID-19. Teach. Teach. Educ..

[22] Callander E., Schofield D. (2016). The impact of poverty on self-efficacy: an Australian longitudinal study. Occup. Med..

[23] Heller A., Altweck L., Hahm S., Michalski N. (2024). The (fe-) male breadwinner? Beliefs about gender roles in East Germany: an age-period-cohort analysis. Thirty Years After Berlin Wall. Routledge.

