# Supplementary material for: General self-efficacy in East and West Germany: A comparison of two German representative cohorts in 2014 and 2022
Source: Public Health Pract (Oxf). 2025 Dec 18;11:100718. doi: 10.1016/j.puhip.2025.100718 (PMC12809091; doi:10.1016/j.puhip.2025.100718)
Supplement: Multimedia component 1 [file mmc1.docx]

**Supplementary materials**

**S1a.** Description of the study procedure

Following a random route procedure, participating households were selected. To contact the identified households, one contact person within each household was randomly selected using the Kish Selection ^1^. For a detailed description of the Kisch Selection, see supplementary material S1b.

The two surveys were carried out as face-to-face household interviews by trained investigators. Inclusion criteria were to be German-speaking and to be at least 14 years old. Prior to the study enrolment, detailed information about the procedures, data collection, and anonymization of personal data were given to the potential participants. Then, verbal informed consent to participate in the study was obtained and confirmed by the interviewers. The studies contents and procedures were approved by the Ethics Commission of the Medical Faculty of the University Leipzig (survey A: 063‐14‐10032014, survey B: 594/21‐ek). Both surveys were conducted in accordance to the ICH-GCP-guidelines and the ICC/ESOMAR International Code of Marketing and Social Research Practice. They were also conducted in accordance with the principles expressed in the Declaration of Helsinki. For detailed description of the response rates, see supplementary material S2a/S2b. Information on plausibility checks can be found in supplementary material S3.

**S1b.** Description of Kish Selection

While the random route procedure enables households to be selected at random, there is often more than one potentially eligible person living within them. Thus, randomly selecting a contact person within a sampled household using the Kish Selection method or other probability methods is essential for maintaining the probability nature of the resulting sample and avoiding systematic bias, ensuring generalisability and enabling inference to be made.

To identify the contact person by using the Kish Selection, all household members aged 16 and over are sorted in descending order by age. A predefined table (Kish grid) is then used to generate a random number indicating which person from the list will be selected for the survey. For more detailed information and comparison of within-household respondent selection methods on demographic representativeness, see ^2^.

Example for the Kish-Selection:


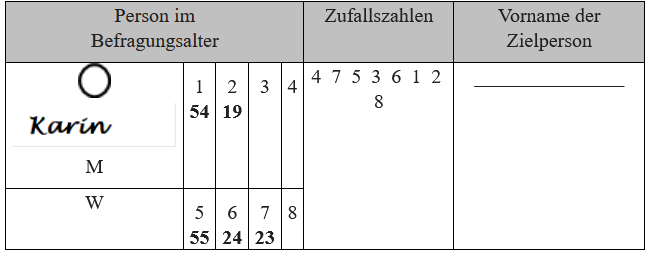


All men living in the household who are at least 16 years old are entered into boxes 1 to 4 in descending order of age. All women are entered into boxes 5 to 8 in the same way. The person whose number appears first in the sequence of random numbers is interviewed, and the order of the random numbers varies in the recording protocols. This means that the target person is selected completely independently of the interviewer and the intermediary contact person. In this example, the target person is the 23-year-old female with the random number 7.

**S2a.** Response rates (survey A)

The following table shows the number of dropouts in relation to the number of interviews conducted.


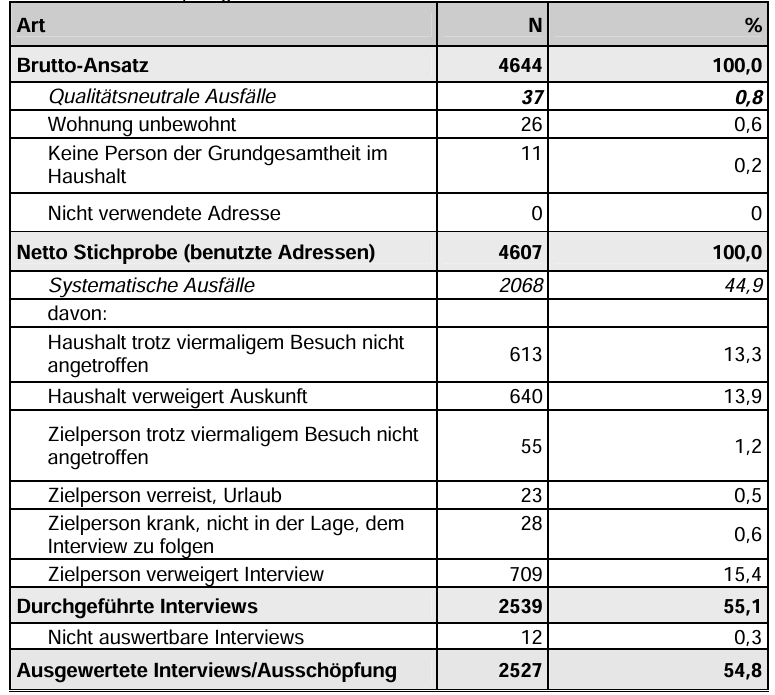


**S2b.** Response rates (survey B)

The following table shows the number of dropouts in relation to the number of interviews conducted.


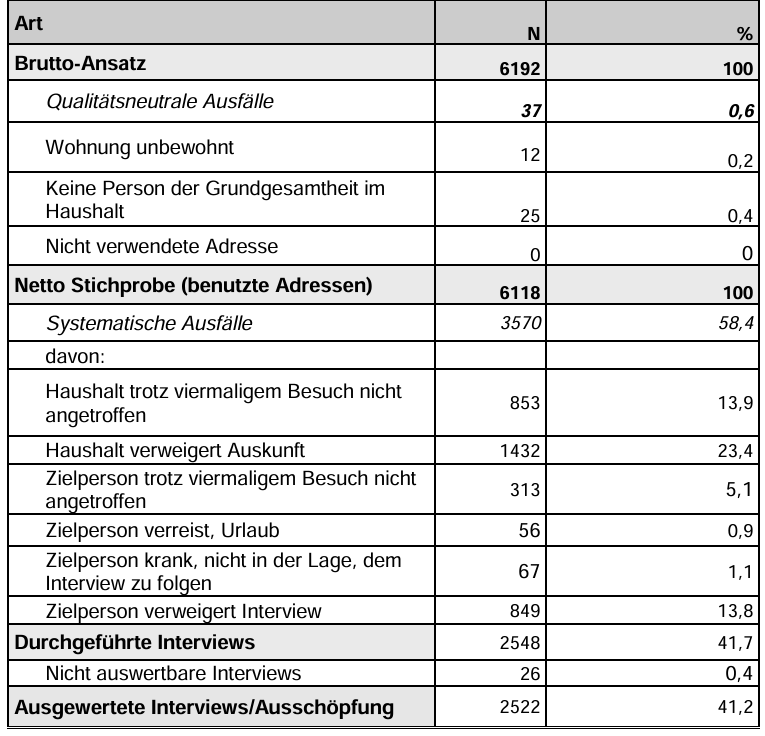


**S3.** Plausibility checks

The data of both surveys was checked for plausibility and formal integrity after it had been collected by error detection programmes. When cleaning up the data also involved referring to the original questionnaires. This was done to ensure that anomalies were not caused by input errors.

**S4.** Results of the tests for homogeneity of variance, the normality of the residuals and the homogeneity of the regression

The assumption of the normality of the residuals could not be met. According to the central limit theorem, however, this violation should not cause major problems. When sample sizes are large enough (>30), parametric procedures can be used even when the data are not normally distributed ^3,4^. Also, the assumption of the homogeneity of the regression slopes was only partly met. To address this issue, the final model included significant interaction terms (age * survey date; age * sex), extending the ANCOVA to a model with non-parallel slopes to better fit the model.

**S4a.** Results of the Levene-Test for analysing homogeneity of variance (ASKU)

| **Variable** | **F - value** | ***df₁*** | ***df_2_*** | **p - value** |
| --- | --- | --- | --- | --- |
| region of residence | 0.25 | 1 | 5,012 | .616 |
| survey date | 3.02 | 1 | 5,012 | .082 |
| sex | 0.67 | 1 | 5,012 | .414 |

***Note.*** *df₁* = degrees of freedom of the group number minus 1; *df₂* = degrees of freedom of the total sample minus the group number

**S4b.** Results of the Levene-Test for analysing homogeneity of variance (Item 1)

| **Variable** | **F - value** | ***df₁*** | ***df_2_*** | **p - value** |
| --- | --- | --- | --- | --- |
| region of residence | 0.12 | 1 | 5,012 | .730 |
| survey date | 1.58 | 1 | 5,012 | .209 |
| sex | 1.94 | 1 | 5,012 | .164 |

***Note.*** *df₁* = degrees of freedom of the group number minus 1; *df₂* = degrees of freedom of the total sample minus the group number

**S4c.** Results of the Levene-Test for analysing homogeneity of variance (Item 2)

| **Variable** | **F - value** | ***df₁*** | ***df_2_*** | **p - value** |
| --- | --- | --- | --- | --- |
| region of residence | 0.00 | 1 | 5,012 | .970 |
| survey date | 2.98 | 1 | 5,012 | .084 |
| sex | 1.89 | 1 | 5,012 | .169 |

***Note.*** *df₁* = degrees of freedom of the group number minus 1; *df₂* = degrees of freedom of the total sample minus the group number

**S4d.** Results of the Levene-Test for analysing homogeneity of variance (Item 3)

| **Variable** | **F - value** | ***df₁*** | ***df_2_*** | **p - value** |
| --- | --- | --- | --- | --- |
| region of residence | 3.68 | 1 | 5,012 | .055 |
| survey date | 2.36 | 1 | 5,012 | .124 |
| sex | 0.05 | 1 | 5,012 | .826 |

***Note.*** *df₁* = degrees of freedom of the group number minus 1; *df₂* = degrees of freedom of the total sample minus the group number

**S5.** Results of the Shapiro-Wilk-Test for analysing normality of the residuals

| **ASKU** | | **Item 1** | | **Item 2** | | **Item 3** | |
| --- | --- | --- | --- | --- | --- | --- | --- |
| **W - value** | **p - value** | **W - value** | **p - value** | **W - value** | **p - value** | **W - value** | **p - value** |
| 0.97 | **< .001** | 0.95 | **< .001** | 0.95 | **< .001** | 0.97 | **< .001** |

***Note.*** significant values are printed in bold

**S6.** Results of the interaction between the covariates (age, education and household income) and the grouping variables (survey date, region of residence and sex) for analysing the homogeneity of the regression slopes

| ​ | **Sum of squares** | ***df*** | **F - value** | **p - value** |
| --- | --- | --- | --- | --- |
| **ASKU** |  |  |  |  |
| **Effect** |  |  |  |  |
| age * region of residence | 0.00 | 1 | 0.01 | .931 |
| education * region of residence | 0.60 | 1 | 0.89 | .345 |
| household income * region of residence | 0.71 | 1 | 1.17 | .279 |
| age * survey date | 3.30 | 1 | 5.36 | **.021** |
| education * survey date | 1.20 | 1 | 2.01 | .156 |
| household income * survey date | 0.04 | 1 | 0.07 | .793 |
| age * sex | 6.90 | 1 | 11.30 | **< .001** |
| education * sex | 1.00 | 1 | 1.62 | .203 |
| household income * sex | 0.51 | 1 | 0.85 | .357 |
| **Item 1** |  |  |  |  |
| **Effect** |  |  |  |  |
| age * region of residence | 0.10 | 1 | 0.16 | .686 |
| education * region of residence | 1.70 | 1 | 2.41 | .121 |
| household income * region of residence | 0.60 | 1 | 0.89 | .346 |
| age * survey date | 2.90 | 1 | 4.08 | **.043** |
| education * survey date | 1.70 | 1 | 2.43 | .119 |
| household income * survey date | 0.00 | 1 | 0.03 | .861 |
| age * sex | 5.90 | 1 | 8.44 | **.004** |
| education * sex | 1.70 | 1 | 2.36 | .125 |
| household income * sex | 0.50 | 1 | 0.79 | .374 |
| **Item 2** |  |  |  |  |
| **Effect** |  |  |  |  |
| age * region of residence | 0.00 | 1 | 0.00 | .992 |
| education * region of residence | 0.40 | 1 | 0.55 | .458 |
| household income * region of residence | 1.60 | 1 | 2.22 | .136 |
| age * survey date | 1.60 | 1 | 2.21 | .137 |
| education * survey date | 0.80 | 1 | 1.08 | .299 |
| household income * survey date | 0.10 | 1 | 0.17 | .676 |
| age * sex | 3.70 | 1 | 5.21 | **.022** |
| education * sex | 0.40 | 1 | 0.50 | .479 |
| household income * sex | 0.50 | 1 | 0.73 | .394 |
| **Item 3** |  |  |  |  |
| **Effect** |  |  |  |  |
| age * region of residence | 0.30 | 1 | 0.37 | .545 |
| education * region of residence | 0.10 | 1 | 0.12 | .733 |
| household income * region of residence | 0.20 | 1 | 0.32 | .573 |
| age * survey date | 6.40 | 1 | 8.08 | **.004** |
| education * survey date | 1.40 | 1 | 1.73 | .189 |
| household income * survey date | 0.00 | 1 | 0.02 | .894 |
| age * sex | 12.60 | 1 | 16.23 | **< .001** |
| education * sex | 1.20 | 1 | 1.58 | .208 |
| household income * sex | 0.50 | 1 | 0.64 | .425 |

***Note.*** *df* = degrees of freedom; significant values are printed in bold

**S7a.** Results of the post-hoc analyses (AV: ASKU; grouping variable: region of residence)

| **survey date** | **sex** | **comparison** | **EMM-Difference** | **SE** | ***df*** | **t - ratio** | **p - value** |
| --- | --- | --- | --- | --- | --- | --- | --- |
| 2014 | male | west vs. east | −0.10 | 0.06 | 4894 | −1.79 | .074 |
| 2022 | female | west vs. east | −0.11 | 0.05 | 4894 | −2.31 | .021 |
| 2014 | male | west vs. east | −0.17 | 0.05 | 4894 | −3.17 | **.002** |
| 2022 | female | west vs. east | 0.05 | 0.06 | 4894 | 0.86 | .393 |

***Note.*** SE = standard error*; df* = degrees of freedom; significant values are printed in bold

**S7b.** Results of the post-hoc analyses (AV: ASKU; grouping variable: survey date)

| **region of residence** | **sex** | **comparison** | **EMM-Difference** | **SE** | ***df*** | **t - ratio** | **p - value** |
| --- | --- | --- | --- | --- | --- | --- | --- |
| west | male | 2014 vs. 2022 | 0.19 | 0.04 | 4894 | 5.12 | **< .001** |
| east | female | 2014 vs. 2022 | 0.17 | 0.07 | 4894 | 2.59 | .010 |
| west | male | 2014 vs. 2022 | 0.12 | 0.03 | 4894 | 3.47 | **< .001** |
| east | female | 2014 vs. 2022 | 0.34 | 0.07 | 4894 | 4.67 | **< .001** |

***Note.*** SE = standard error*; df* = degrees of freedom; significant values are printed in bold

**S7c.** Results of the post-hoc analyses (AV: ASKU; grouping variable: sex)

| **region of residence** | **survey date** | **comparison** | **EMM-Difference** | **SE** | ***df*** | **t -ratio** | **p - value** |
| --- | --- | --- | --- | --- | --- | --- | --- |
| west | 2014 | male vs. female | 0.17 | 0.04 | 4894 | 4.84 | **< .001** |
| east | 2014 | male vs. female | 0.10 | 0.07 | 4894 | 1.48 | .140 |
| west | 2022 | male vs. female | 0.10 | 0.04 | 4894 | 2.90 | **.004** |
| east | 2022 | male vs. female | 0.27 | 0.07 | 4894 | 3.88 | **< .001** |

***Note.*** SE = standard error*; df* = degrees of freedom; significant values are printed in bold

**S7d.** Results of the post-hoc analyses (AV: Item 1; grouping variable: region of residence)

| **survey date** | **sex** | **comparison** | **EMM-Difference** | **SE** | ***df*** | **t - ratio** | **p -value** |
| --- | --- | --- | --- | --- | --- | --- | --- |
| 2014 | male | west vs. east | -0.15 | 0.0612 | 4894 | -2.481 | **.013** |
| 2022 | male | west vs. east | -0.16 | 0.0529 | 4894 | -3.033 | **.002** |
| 2014 | female | west vs. east | -0.18 | 0.0569 | 4894 | -3.176 | **.002** |
| 2022 | female | west vs. east | -0.02 | 0.0628 | 4894 | -0.358 | .720 |

***Note.*** SE = standard error*; df* = degrees of freedom; significant values are printed in bold

**S7e.** Results of the post-hoc analyses (AV: Item 1; grouping variable: survey date)

| **region of residence** | **sex** | **comparison** | **EMM-Difference** | **SE** | ***df*** | **t - ratio** | **p - value** |
| --- | --- | --- | --- | --- | --- | --- | --- |
| west | male | 2014 vs. 2022 | 0.23 | 0.04 | 4894 | 5.88 | **< .001** |
| east | male | 2014 vs. 2022 | 0.22 | 0.07 | 4894 | 3.07 | **.002** |
| west | female | 2014 vs. 2022 | 0.18 | 0.04 | 4894 | 4.88 | **< .001** |
| east | female | 2014 vs. 2022 | 0.34 | 0.08 | 4894 | 4.36 | **< .001** |

***Note.*** SE = standard error*; df* = degrees of freedom; significant values are printed in bold

**S7f.** Results of the post-hoc analyses (AV: Item 1; grouping variable: sex)

| **region of residence** | **survey date** | **comparison** | **EMM-Difference** | **SE** | ***df*** | **t - ratio** | **p - value** |
| --- | --- | --- | --- | --- | --- | --- | --- |
| west | 2014 | male vs. female | 0.15 | 0.04 | 4894 | 4.06 | **< .001** |
| east | 2014 | male vs. female | 0.12 | 0.07 | 4894 | 1.65 | .099 |
| west | 2022 | male vs. female | 0.10 | 0.04 | 4894 | 2.70 | **.007** |
| east | 2022 | male vs. female | 0.24 | 0.07 | 4894 | 3.26 | **.001** |

***Note.*** SE = standard error*; df* = degrees of freedom; significant values are printed in bold

**S7g.** Results of the post-hoc analyses (AV: Item 2; grouping variable: region of residence)

| **survey date** | **sex** | **comparison** | **EMM-Difference** | **SE** | ***df*** | **t - ratio** | **p -value** |
| --- | --- | --- | --- | --- | --- | --- | --- |
| 2014 | male | west vs. east | -0.11 | 0.06 | 4895 | -1.74 | .083 |
| 2022 | male | west vs. east | -0.80 | 0.05 | 4895 | -1.50 | .135 |
| 2014 | female | west vs. east | -0.19 | 0.06 | 4895 | -3.26 | **.001** |
| 2022 | female | west vs. east | 0.05 | 0.06 | 4895 | 0.86 | .391 |

***Note.*** SE = standard error*; df* = degrees of freedom; significant values are printed in bold

**S7h.** Results of the post-hoc analyses (AV: Item 2; grouping variable: survey date)

| **region of residence** | **sex** | **comparison** | **EMM-Difference** | **SE** | ***df*** | **t - ratio** | **p - value** |
| --- | --- | --- | --- | --- | --- | --- | --- |
| west | male | 2014 vs. 2022 | 0.16 | 0.04 | 4895 | 3.98 | **< .001** |
| east | male | 2014 vs. 2022 | 0.18 | 0.07 | 4895 | 2.54 | **.011** |
| west | female | 2014 vs. 2022 | 0.11 | 0.04 | 4895 | 3.11 | **.002** |
| east | female | 2014 vs. 2022 | 0.35 | 0.08 | 4895 | 4.58 | **< .001** |

***Note.*** SE = standard error*; df* = degrees of freedom; significant values are printed in bold

**S7i.** Results of the post-hoc analyses (AV: Item 2; grouping variable: sex)

| **region of residence** | **survey date** | **comparison** | **EMM-Difference** | **SE** | ***df*** | **t - ratio** | **p - value** |
| --- | --- | --- | --- | --- | --- | --- | --- |
| west | 2014 | male vs. female | 0.17 | 0.04 | 4895 | 4.60 | **< .001** |
| east | 2014 | male vs. female | 0.09 | 0.08 | 4895 | 1.24 | .215 |
| west | 2022 | male vs. female | 0.13 | 0.04 | 4895 | 3.49 | **< .001** |
| east | 2022 | male vs. female | 0.27 | 0.07 | 4895 | 3.60 | **< .001** |

***Note.*** SE = standard error*; df* = degrees of freedom; significant values are printed in bold

**S7j.** Results of the post-hoc analyses (AV: Item 3; grouping variable: region of residence)

| **survey date** | **sex** | **comparison** | **EMM-Difference** | **SE** | ***df*** | **t - ratio** | **p -value** |
| --- | --- | --- | --- | --- | --- | --- | --- |
| 2014 | male | west vs. east | -0.04 | 0.06 | 4894 | -0.65 | .519 |
| 2022 | male | west vs. east | -0.10 | 0.06 | 4894 | -1.84 | .066 |
| 2014 | female | west vs. east | -0.13 | 0.06 | 4894 | -2.23 | **.026** |
| 2022 | female | west vs. east | 0.12 | 0.06 | 4894 | 1.75 | .080 |

***Note.*** SE = standard error*; df* = degrees of freedom; significant values are printed in bold

**S7k.** Results of the post-hoc analyses (AV: Item 3; grouping variable: survey date)

| **region of residence** | **sex** | **comparison** | **EMM-Difference** | **SE** | ***df*** | **t - ratio** | **p - value** |
| --- | --- | --- | --- | --- | --- | --- | --- |
| west | male | 2014 vs. 2022 | 0.17 | 0.04 | 4894 | 4.27 | **< .001** |
| east | male | 2014 vs. 2022 | 0.11 | 0.08 | 4894 | 1.50 | .133 |
| west | female | 2014 vs. 2022 | 0.06 | 0.04 | 4894 | 1.63 | .104 |
| east | female | 2014 vs. 2022 | 0.31 | 0.08 | 4894 | 3.85 | **< .001** |

***Note.*** SE = standard error*; df* = degrees of freedom; significant values are printed in bold

**S7l.** Results of the post-hoc analyses (AV: Item 3; grouping variable: sex)

| **region of residence** | **survey date** | **comparison** | **EMM-Difference** | **SE** | ***df*** | **t - ratio** | **p - value** |
| --- | --- | --- | --- | --- | --- | --- | --- |
| west | 2014 | male vs. female | 0.18 | 0.04 | 4894 | 4.66 | **< .001** |
| east | 2014 | male vs. female | 0.09 | 0.08 | 4894 | 1.16 | .245 |
| west | 2022 | male vs. female | 0.07 | 0.04 | 4894 | 1.82 | .069 |
| east | 2022 | male vs. female | 0.29 | 0.08 | 4894 | 3.76 | **< .001** |

***Note.*** SE = standard error*; df* = degrees of freedom; significant values are printed in bold

**References:**

1. Kish L. A Procedure for Objective Respondent Selection within the Household. *Journal of the American Statistical Association*. 1949;44:380-387.

2. Yan T, Tourangeau R, McAloon R. A Meta-analysis of within-household respondent selection methods on demographic representativeness. 2015.

3. Arsham DH. Systems Simulation: The Shortest Route to Applications. *National Science Foundation*. 2020.

4. Hays WL. *Statistics*. vol 5th Edition. Harcourt Brace College Publishers; 1994.
